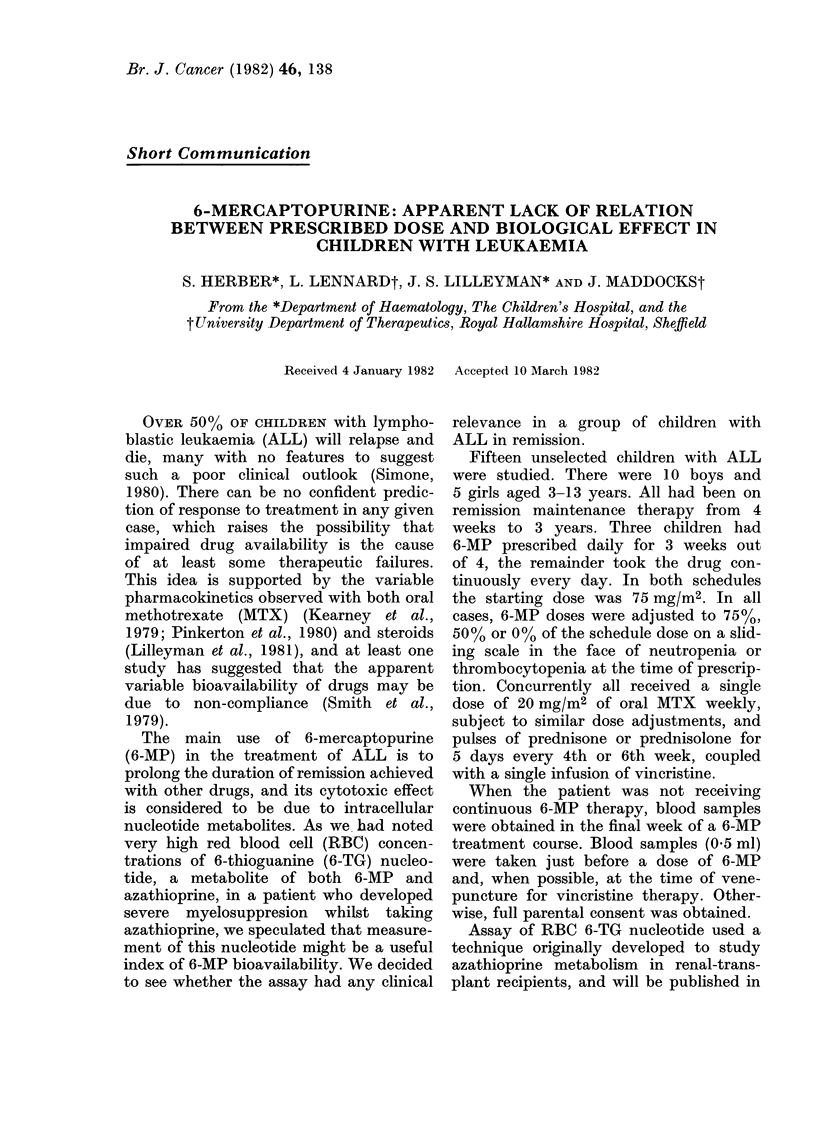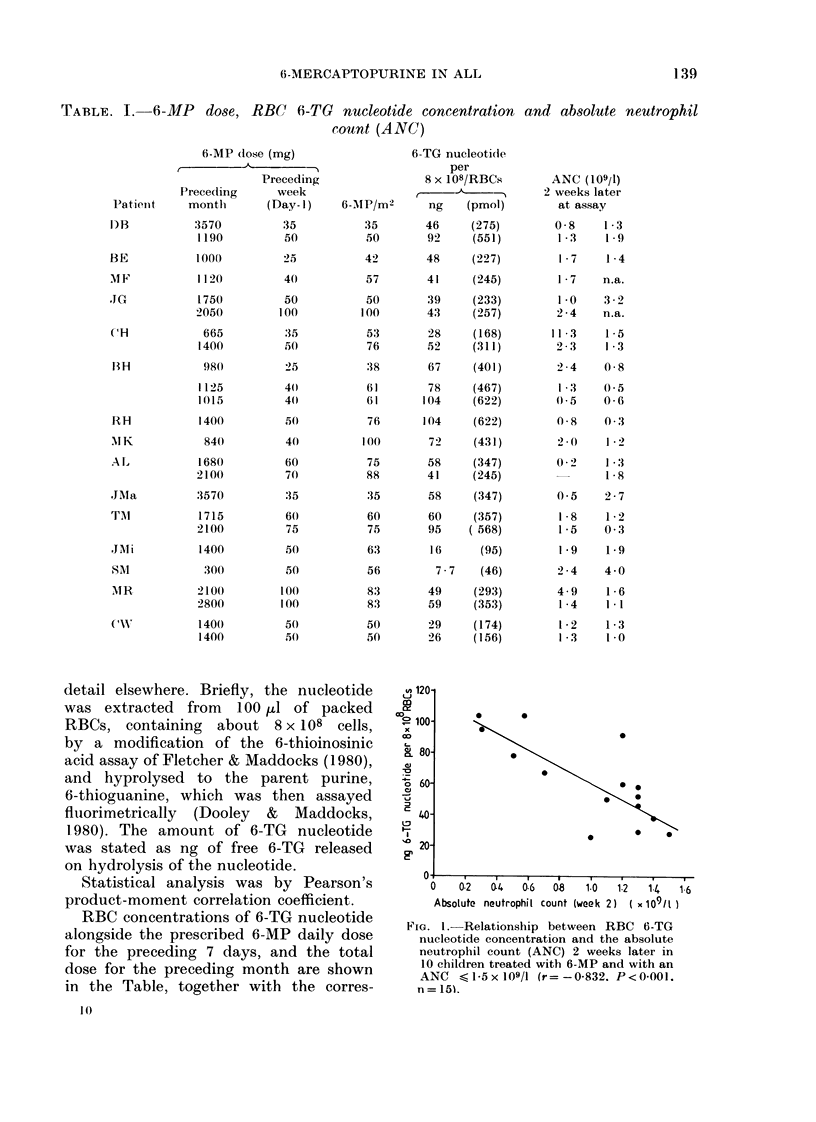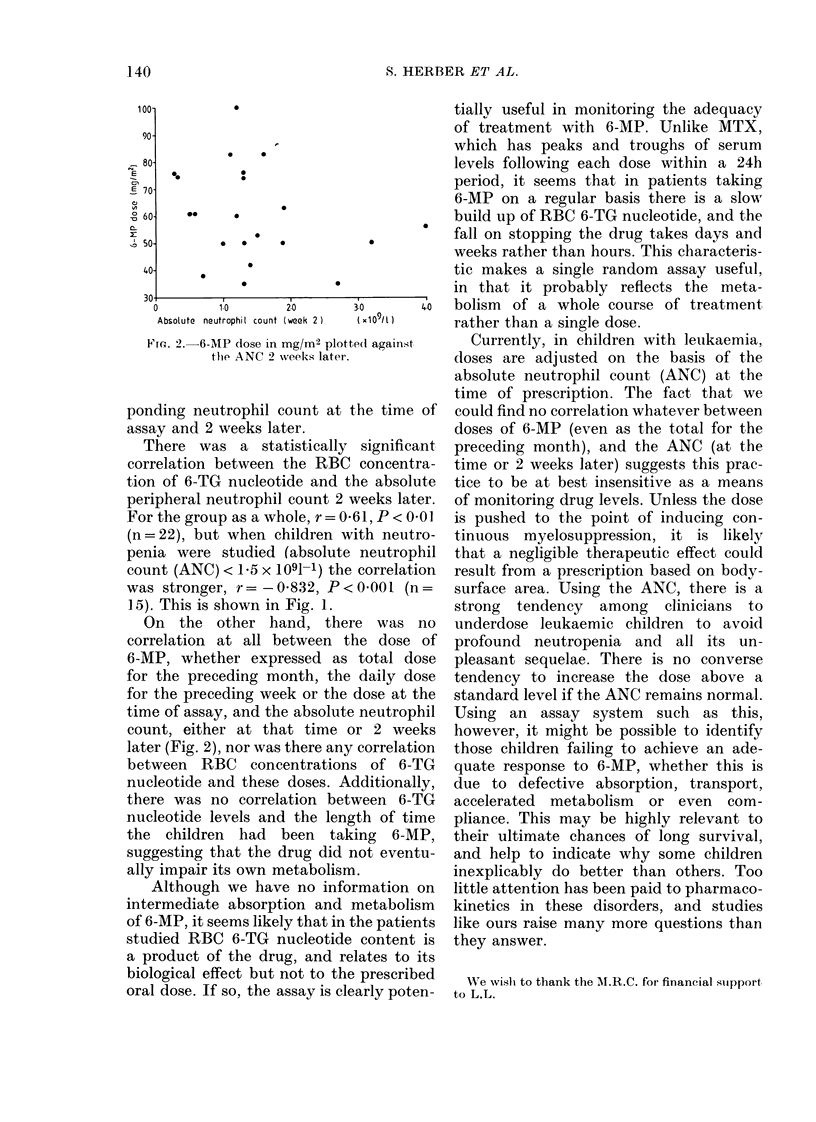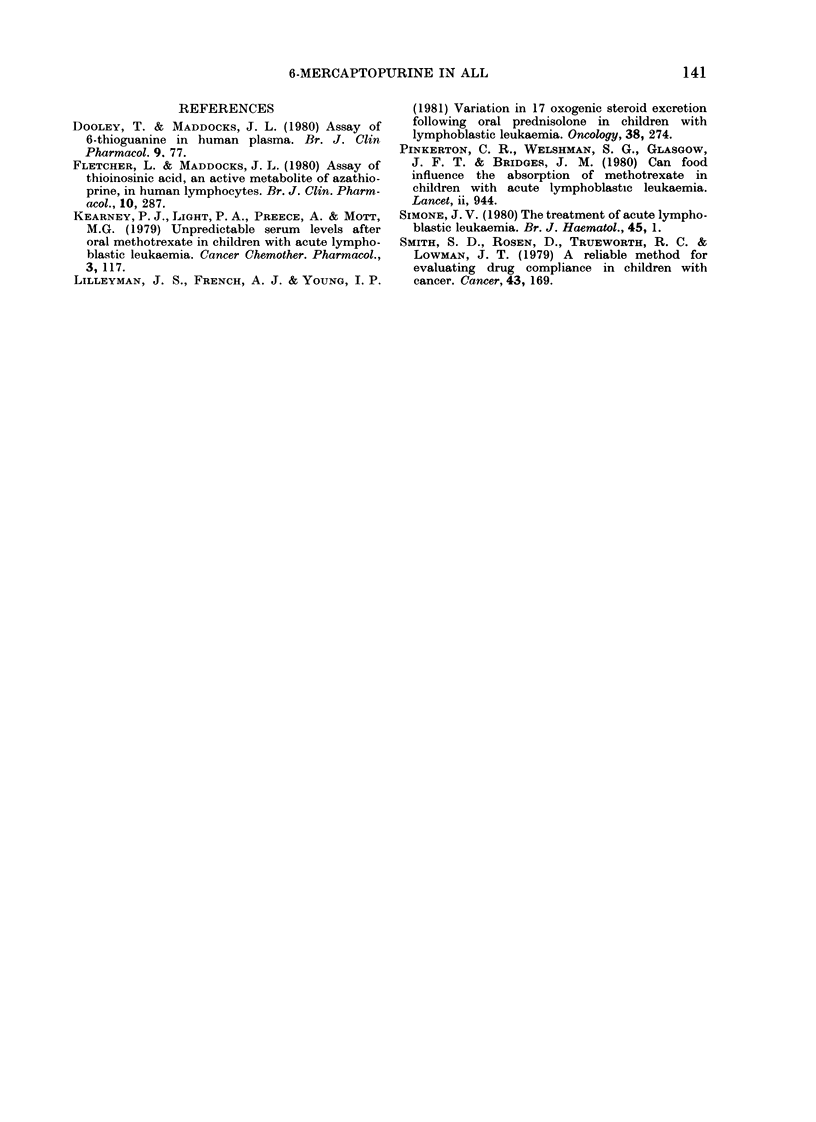# 6-Mercaptopurine: apparent lack of relation between prescribed dose and biological effect in children with leukaemia.

**DOI:** 10.1038/bjc.1982.175

**Published:** 1982-07

**Authors:** S. Herber, L. Lennard, J. S. Lilleyman, J. Maddocks


*Br. J. Cancer* (1982) **46**, 138

***Short Communication***

### 6-MERCAPTOPURINE: APPARENT LACK OF RELATION BETWEEN PRESCRIBED DOSE AND BIOLOGICAL EFFECT IN CHILDREN WITH LEUKAEMIA

S. HERBER*, L. LENNARD†, J. S. LILLEYMAN* AND J. MADDOCKS†

*From the *Department of Haematology, The Children's Hospital, and the †University Department of Therapeutics, Royal Hallamshire Hospital, Sheffield*

Received 4 January 1982 Accepted 10 March 1982

OVER 50% OF CHILDREN with lymphoblastic leukaemia (ALL) will relapse and die, many with no features to suggest such a poor clinical outlook (Simone, 1980). There can be no confident prediction of response to treatment in any given case, which raises the possibility that impaired drug availability is the cause of at least some therapeutic failures. This idea is supported by the variable pharmacokinetics observed with both oral methotrexate (MTX) (Kearney *et al.*, 1979; Pinkerton *et al.*, 1980) and steroids (Lilleyman *et al.*, 1981), and at least one study has suggested that the apparent variable bioavailability of drugs may be due to non-compliance (Smith *et al.*, 1979).

The main use of 6-mercaptopurine (6-MP) in the treatment of ALL is to prolong the duration of remission achieved with other drugs, and its cytotoxic effect is considered to be due to intracellular nucleotide metabolites. As we had noted very high red blood cell (RBC) concentrations of 6-thioguanine (6-TG) nucleotide, a metabolite of both 6-MP and azathioprine, in a patient who developed severe myelosuppresion whilst taking azathioprine, we speculated that measurement of this nucleotide might be a useful index of 6-MP bioavailability. We decided to see whether the assay had any clinical relevance in a group of children with ALL in remission.

Fifteen unselected children with ALL were studied. There were 10 boys and 5 girls aged 3–13 years. All had been on remission maintenance therapy from 4 weeks to 3 years. Three children had 6-MP prescribed daily for 3 weeks out of 4, the remainder took the drug continuously every day. In both schedules the starting dose was 75 mg/m$^2$. In all cases, 6-MP doses were adjusted to 75%, 50% or 0% of the schedule dose on a sliding scale in the face of neutropenia or thrombocytopenia at the time of prescription. Concurrently all received a single dose of 20 mg/m$^2$ of oral MTX weekly, subject to similar dose adjustments, and pulses of prednisone or prednisolone for 5 days every 4th or 6th week, coupled with a single infusion of vincristine.

When the patient was not receiving continuous 6-MP therapy, blood samples were obtained in the final week of a 6-MP treatment course. Blood samples (0·5 ml) were taken just before a dose of 6-MP and, when possible, at the time of venepuncture for vincristine therapy. Otherwise, full parental consent was obtained.

Assay of RBC 6-TG nucleotide used a technique originally developed to study azathioprine metabolism in renal-transplant recipients, and will be published in

TABLE. I.—*6-MP dose, RBC 6-TG nucleotide concentration and absolute neutrophil count (ANC)*

| Patient | 6-MP dose (mg) Preceding month | Preceding week (Day-1) | 6-MP/m² | 6-TG nucleotide per 8 x 10⁸/RBCs ng | (pmol) | ANC (10⁹/l) 2 weeks later | at assay |
|---|---|---|---|---|---|---|---|
| DB | 3570 | 35 | 35 | 46 | (275) | 0·8 | 1·3 |
| | 1190 | 50 | 50 | 92 | (551) | 1·3 | 1·9 |
| BE | 1000 | 25 | 42 | 48 | (227) | 1·7 | 1·4 |
| MF | 1120 | 40 | 57 | 41 | (245) | 1·7 | n.a. |
| JG | 1750 | 50 | 50 | 39 | (233) | 1·0 | 3·2 |
| | 2050 | 100 | 100 | 43 | (257) | 2·4 | n.a. |
| CH | 665 | 35 | 53 | 28 | (168) | 11·3 | 1·5 |
| | 1400 | 50 | 76 | 52 | (311) | 2·3 | 1·3 |
| BH | 980 | 25 | 38 | 67 | (401) | 2·4 | 0·8 |
| | 1125 | 40 | 61 | 78 | (467) | 1·3 | 0·5 |
| | 1015 | 40 | 61 | 104 | (622) | 0·5 | 0·6 |
| RH | 1400 | 50 | 76 | 104 | (622) | 0·8 | 0·3 |
| MK | 840 | 40 | 100 | 72 | (431) | 2·0 | 1·2 |
| AL | 1680 | 60 | 75 | 58 | (347) | 0·2 | 1·3 |
| | 2100 | 70 | 88 | 41 | (245) | — | 1·8 |
| JMa | 3570 | 35 | 35 | 58 | (347) | 0·5 | 2·7 |
| TM | 1715 | 60 | 60 | 60 | (357) | 1·8 | 1·2 |
| | 2100 | 75 | 75 | 95 | ( 568) | 1·5 | 0·3 |
| JMi | 1400 | 50 | 63 | 16 | (95) | 1·9 | 1·9 |
| SM | 300 | 50 | 56 | 7·7 | (46) | 2·4 | 4·0 |
| MR | 2100 | 100 | 83 | 49 | (293) | 4·9 | 1·6 |
| | 2800 | 100 | 83 | 59 | (353) | 1·4 | 1·1 |
| CW | 1400 | 50 | 50 | 29 | (174) | 1·2 | 1·3 |
| | 1400 | 50 | 50 | 26 | (156) | 1·3 | 1·0 |

detail elsewhere. Briefly, the nucleotide was extracted from 100 μl of packed RBCs, containing about $8 \times 10^8$ cells, by a modification of the 6-thioinosinic acid assay of Fletcher & Maddocks (1980), and hyprolysed to the parent purine, 6-thioguanine, which was then assayed fluorimetrically (Dooley & Maddocks, 1980). The amount of 6-TG nucleotide was stated as ng of free 6-TG released on hydrolysis of the nucleotide.

Statistical analysis was by Pearson's product-moment correlation coefficient.

RBC concentrations of 6-TG nucleotide alongside the prescribed 6-MP daily dose for the preceding 7 days, and the total dose for the preceding month are shown in the Table, together with the corres-

FIG. 1.—Relationship between RBC 6-TG nucleotide concentration and the absolute neutrophil count (ANC) 2 weeks later in 10 children treated with 6-MP and with an ANC $\leq 1{\cdot}5 \times 10^9$/l ($r = -0{\cdot}832$, $P < 0{\cdot}001$, n = 15).

FIG. 2.—6-MP dose in mg/m² plotted against the ANC 2 weeks later.

ponding neutrophil count at the time of assay and 2 weeks later.

There was a statistically significant correlation between the RBC concentration of 6-TG nucleotide and the absolute peripheral neutrophil count 2 weeks later. For the group as a whole, $r = 0{\cdot}61, P < 0{\cdot}01$ ($n = 22$), but when children with neutropenia were studied (absolute neutrophil count (ANC) $< 1{\cdot}5 \times 10^9 l^{-1}$) the correlation was stronger, $r = -0{\cdot}832$, $P < 0{\cdot}001$ ($n = 15$). This is shown in Fig. 1.

On the other hand, there was no correlation at all between the dose of 6-MP, whether expressed as total dose for the preceding month, the daily dose for the preceding week or the dose at the time of assay, and the absolute neutrophil count, either at that time or 2 weeks later (Fig. 2), nor was there any correlation between RBC concentrations of 6-TG nucleotide and these doses. Additionally, there was no correlation between 6-TG nucleotide levels and the length of time the children had been taking 6-MP, suggesting that the drug did not eventually impair its own metabolism.

Although we have no information on intermediate absorption and metabolism of 6-MP, it seems likely that in the patients studied RBC 6-TG nucleotide content is a product of the drug, and relates to its biological effect but not to the prescribed oral dose. If so, the assay is clearly potentially useful in monitoring the adequacy of treatment with 6-MP. Unlike MTX, which has peaks and troughs of serum levels following each dose within a 24h period, it seems that in patients taking 6-MP on a regular basis there is a slow build up of RBC 6-TG nucleotide, and the fall on stopping the drug takes days and weeks rather than hours. This characteristic makes a single random assay useful, in that it probably reflects the metabolism of a whole course of treatment rather than a single dose.

Currently, in children with leukaemia, doses are adjusted on the basis of the absolute neutrophil count (ANC) at the time of prescription. The fact that we could find no correlation whatever between doses of 6-MP (even as the total for the preceding month), and the ANC (at the time or 2 weeks later) suggests this practice to be at best insensitive as a means of monitoring drug levels. Unless the dose is pushed to the point of inducing continuous myelosuppression, it is likely that a negligible therapeutic effect could result from a prescription based on body-surface area. Using the ANC, there is a strong tendency among clinicians to underdose leukaemic children to avoid profound neutropenia and all its unpleasant sequelae. There is no converse tendency to increase the dose above a standard level if the ANC remains normal. Using an assay system such as this, however, it might be possible to identify those children failing to achieve an adequate response to 6-MP, whether this is due to defective absorption, transport, accelerated metabolism or even compliance. This may be highly relevant to their ultimate chances of long survival, and help to indicate why some children inexplicably do better than others. Too little attention has been paid to pharmacokinetics in these disorders, and studies like ours raise many more questions than they answer.

We wish to thank the M.R.C. for financial support to L.L.

### REFERENCES

DOOLEY, T. & MADDOCKS, J. L. (1980) Assay of 6-thioguanine in human plasma. *Br. J. Clin Pharmacol.* **9**, 77.

FLETCHER, L. & MADDOCKS, J. L. (1980) Assay of thioinosinic acid, an active metabolite of azathioprine, in human lymphocytes. *Br. J. Clin. Pharmacol.*, **10**, 287.

KEARNEY, P. J., LIGHT, P. A., PREECE, A. & MOTT, M.G. (1979) Unpredictable serum levels after oral methotrexate in children with acute lymphoblastic leukaemia. *Cancer Chemother. Pharmacol.*, **3**, 117.

LILLEYMAN, J. S., FRENCH, A. J. & YOUNG, I. P. (1981) Variation in 17 oxogenic steroid excretion following oral prednisolone in children with lymphoblastic leukaemia. *Oncology*, **38**, 274.

PINKERTON, C. R., WELSHMAN, S. G., GLASGOW, J. F. T. & BRIDGES, J. M. (1980) Can food influence the absorption of methotrexate in children with acute lymphoblastic leukaemia. *Lancet*, ii, 944.

SIMONE, J. V. (1980) The treatment of acute lymphoblastic leukaemia. *Br. J. Haematol.*, **45**, 1.

SMITH, S. D., ROSEN, D., TRUEWORTH, R. C. & LOWMAN, J. T. (1979) A reliable method for evaluating drug compliance in children with cancer. *Cancer*, **43**, 169.